# Prevalence and characterization of heart failure in Aragon, Spain (ICAR study)

**DOI:** 10.3389/fcvm.2026.1749081

**Published:** 2026-03-18

**Authors:** Juan Carlos Romero-Vigara, Jose Ignacio González Lillo, David Bierge Valero, Patricia Arbués-Espinosa, Sandra Luz-Miguel, Jose Ramón Garcia-Solans, Irantzu Bengoa-Urrengoechea, Susana Larripa de la Natividad, Mónica Salazar González, Elisa Pilar Salazar González, Francisco Manuel Adán-Gil

**Affiliations:** 1 Primary Care, Health Service of Aragon, Zaragoza, Spain; 2 Primary Care, IISA, Aragon Health Research Institute, Zaragoza, Spain; 3 Cardiology, Health Service of Aragon, Zaragoza, Spain; 4 Primary Care/Preventive Medicine and Public Health, Health Service of La Rioja, Logroño, Spain

**Keywords:** comorbidity, drug therapy, heart failure, population surveillance, prevalence

## Abstract

**Introduction:**

Heart failure (HF) imposes a significant clinical and care burden. The ICAR study sought to address the absence of recent population estimates and an integrated approach to the diagnosis of HF in Spain, specifically in the region of Aragon.

**Methods:**

Population-based, observational, retrospective, descriptive cross-sectional study conducted over a 5-year period (2019–2023) in Aragon. It included individuals residing in the region aged ≥18 years with HF and was based on BIGAN database.

**Results:**

We identified 30 677 individuals with HF (prevalence 2.7%). Prevalence increased with age (>75 years, 72.3%) and was higher in women (2.8% vs. 2.6%). Mean time since diagnosis was 6.6 years (standard deviation: 5.9). The main comorbidities were hypertension (63.5%); dyslipidemia (48.9%); obesity (39.5%); chronic kidney disease (35.4%); atrial fibrillation (34.0%); diabetes (32.3%); and anemia (30.6%). Diagnosis was primarily hospital-based: ∼33% were diagnosed exclusively during the hospital stay, ∼20% in primary care, and ∼45% across different levels of care. We observed a more than four-fold increase in hospital admissions in patients with HF and an approximately 45% increase in emergency visits for HF between 2019 and 2023. The main treatments prescribed were loop diuretics (57.5%), while the most common for HF were beta-blockers (47.4%), angiotensin receptor blockers (37.0%), mineralocorticoid receptor antagonists (23.5%), sodium-glucose cotransporter type 2 inhibitors (22.5%), angiotensin-converting enzyme inhibitors (17.7%), and sacubitril/valsartan (8.9%).

**Conclusion:**

HF in Aragon shows high prevalence and complexity in an aging population with multiple pathologies, in the framework of a reactive care model characterized by in-hospital confirmation and rising resource utilization.

## Graphical Abstract

**Figure:**
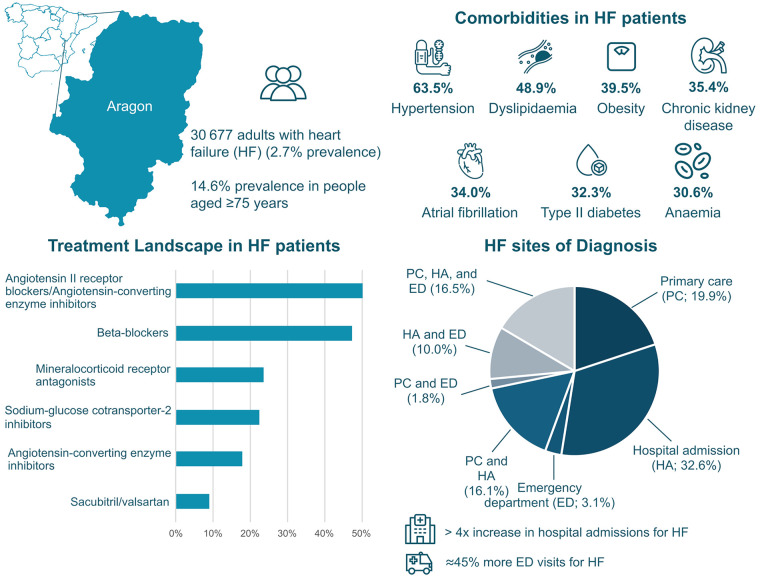

## 1 Introduction

Heart failure (HF) is a major global public health concern (1). This complex clinical syndrome stems from a structural and/or functional cardiac abnormality and is characterized by symptom progression leading to a high rate of comorbidity, frequent hospital admissions (HA), deterioration in quality of life, poor prognosis, and a high mortality rate, as well as considerable healthcare resource utilization (2–5).

Recent decades have seen an increase in the prevalence of HF worldwide, with the global number of cases increasing by 91.9% from 1990 to 2017 (1). However, the data available are usually from partial population cohorts, and few studies have evaluated prevalence based on the population data from an entire country.

In Spain, the estimated prevalence of HF varies between 1% and 7% according to the studies published to date (3, 6, 7). The Spanish observational, retrospective, population-based HF-PATHWAYS study estimated a prevalence of 1.89% in individuals aged ≥18 years in 2019 (7), while the observational, prospective, multicenter IBERICAN study conducted in adults ≥18 years reported an estimated prevalence of 3.1% (8). These results are consistent with those described in European countries such as Sweden, the United Kingdom, Portugal and Germany (9). Additionally, the PRICE demographic study, which included data from 15 hospitals and 55 health centers in Spain during 2004 and 2005, reported a prevalence of congestive HF of 6.8% in the population aged ≥45 years (6). HF is among the leading causes of HA in individuals aged ≥65 years in Spain, accounting for around 3% of all HAs (2) and 2.5% of healthcare costs (3).

One of the main reasons behind the increased prevalence and incidence of HF in recent decades is population ageing driven by increased survival in patients with chronic diseases and improved care (6, 10). To this clinical complexity are added social and geriatric factors that negatively affect prognosis. Recent studies have identified frailty as an independent predictor of mortality, readmissions, and functional deterioration in older patients with HF (11, 12), and an unfavorable social profile has been identified as an independent risk factor for mortality in patients with advanced HF (13). The presence of certain chronic diseases has also been reported as a risk factor for the onset of HF. According to a number of studies conducted in Spain, 22.4% of patients with type 2 diabetes mellitus (T2DM) with no previous cardiovascular disease developed HF (14), and 38.7% of hospital discharges for HF occur in patients with T2DM (15). Moreover, a population study in Catalonia (Spain) showed that individuals with T2DM were admitted to hospital for an episode of HF more frequently than non-diabetic patients (4.7% vs. 1.7%, respectively) (16).

Notwithstanding improvements in the diagnosis and treatment (pharmacological and non-pharmacological) of HF both in patients with preserved (HFpEF) and reduced (HFrEF) ejection fraction, early detection and appropriate follow-up remain a priority, especially within the primary care (PC) setting (17). Establishing inter-level coordination strategies that enhance the quality and continuity of care for chronic patients and implementing effective preventive interventions will address this challenge and reduce HAs for HF and their associated economic burden (18).

In this context, it is essential to have epidemiological data to better characterize patients with HF, to identify opportunities for improving care, and to guide health policies. To this end, the main objective of this study was to estimate the prevalence and evolution of HF in the Autonomous Region of Aragon, Spain. The secondary objectives were to describe the most relevant demographic and clinical characteristics of the diagnosed patients, and to analyze the use of healthcare resources associated with decompensated HF, including visits to the emergency department (ED) and HAs.

## 2 Methods

### 2.1 Study design

This was a retrospective, population-based descriptive study with patients diagnosed with HF in the Autonomous Region of Aragon (Spain), leveraging repeated cross-sectional snapshots between 2019 and 2023, and with a longitudinal description of emergency department visits and hospitalizations for heart failure. The study followed a pre-specified protocol (version 14-02-24) that establishes the study criteria, the operational definition of variables, and the analytical plan. The study was evaluated and authorized by the Aragon Clinical Research Ethics Committee (CEICA) on 6 March 2024 (C.I. PI24/095). Reporting adhered to the STROBE statement for observational studies and complies with the minimum CODE-EHR standards.

### 2.2 Study population

The study population included all residents in Aragon ≥18 years assigned to the Health Service of Aragon and alive as of 31 December 2023, with evidence of a diagnosis of HF at any time prior to the index date (between 2019 and 2023). Individuals who did not meet these criteria were excluded.

The index date was defined as the first record of any of the specified HF codes between 2019 and 2023. Patients diagnosed with HF prior to the study period were also included, provided this diagnosis continued to be recorded in the medical record in January 2019. Subsequent records were used to describe healthcare contacts occurring between 2019 and 2023 at the population level.

### 2.3 Data sources

The data were obtained from the BIGAN Health Information Platform (19), which includes healthcare information routinely generated between January 2019 and December 2023 at all levels of the public health system (PC, ED, and HA). This platform consolidates the medical records generated during the treatment of patients seen by the National Health System, which covers more than 90% of the population of Aragon. Access to the database was evaluated and accepted by the Aragon Institute of Health Sciences.

### 2.4 Definition of heart failure

HF was identified by the presence of ICPC-2 code K77 in PC records and ICD-10 code I50 (any subcategory) in hospital and ED records.

Because echocardiography reports were inaccessible to automated analysis, the HFrEF and HFpEF phenotypes could not be differentiated, so HF was analyzed as a single entity.

There were also considerable under-reporting and temporal variability in the functional class as defined by the New York Heart Association (NYHA) classification, so this field was also excluded from the analysis.

### 2.5 Study variables

The main variable was the diagnosis of HF and the time since diagnosis.

Demographic variables included age and sex.

Clinical variables included comorbidities such as chronic kidney disease (CKD), hypertension (HTN), dyslipidemia, T2DM, smoking, alcoholism, obesity, thyroid disorders (hyper- and hypothyroidism), iron deficiency anemia, cirrhosis, ischemic heart disease, vascular disease, atrial fibrillation, asthma, and chronic obstructive pulmonary disease (COPD). For each variable, the measurement performed during 2023 that was closest to 31 December 2023 was considered. All clinical variables were identified in the database using the International Classification of Primary Care, 2nd edition (ICPC-2, Supplementary Table S1).

To establish the presence of undiagnosed CKD and to avoid false positives in patients without a recorded diagnosis of CKD [whether in PC (ICPC-2 code U99), hospital, or ED records], we reviewed the estimated glomerular filtration rate (eGFR) values (eGFR <60 mL/min/1.73 m^2^) or, if absent, plasma creatinine (pCr) values (cut-off values in men, 1.3 mg/dL; in women, 1.1 mg/dL) recorded between 1 January 2019 and 31 December 2023. In patients in whom there was no recorded diagnosis of CKD and the eGFR could not be determined, the urine albumin-creatinine ratio (UACR) (>30 mg/g) from 1 January 2019 to 31 December 2023 was considered. In both cases, the diagnosis of CKD was established if two values supporting the diagnosis recorded at least 3 months apart were available. In the case of obesity, in addition to the cases coded in PC, cases were also classified based on the body mass index (BMI) data ≥30 kg/m^2^ closest to 31 December 2023 in the medical record.

The following laboratory parameters were also analyzed: pCr, natriuretic peptides (BNP and proBNP), hemoglobin, platelet count, ferritin, thyroid-stimulating hormone (TSH), aspartate aminotransferase (AST), alanine aminotransferase (ALT), alkaline phosphatase (ALP), and glycated hemoglobin (HbA1c). For all parameters, the measurement performed during 2023 that was closest to 31 December 2023 was considered.

Active treatments as of 31 December 2023 were identified, which included drugs indicated for HF such as angiotensin-converting enzyme inhibitors (ACEI), angiotensin II receptor blockers (ARB), angiotensin receptor-neprilysin inhibitors (ARNI), diuretics, beta-blockers, mineralocorticoid receptor antagonists (MRA), ivabradine, and organic nitrates. Glucose-lowering treatments, sodium-glucose cotransporter-2 inhibitors (SGLT2i), lipid-lowering agents, and anticoagulants were also included. All pharmacological treatments were identified in the database using the Anatomical Therapeutic Chemical classification (ATC, Supplementary Table S2).

ED visits and HA for decompensated HF were collected (2019 to 2023), and the level of care (PC, ED, or HA) in which the HF was first diagnosed between 2019 and 2023was recorded.

### 2.6 Statistical analysis

Qualitative variables are described by absolute frequencies and percentages. Quantitative variables are summarized using measures of central tendency (mean or median) and dispersion: mean and standard deviation (SD) or, where appropriate, median and interquartile range (IQR). The differences described in the text are strictly numerical; their statistical significance was not evaluated and no hypothesis testing was performed. Statistical analysis was performed using R software (version 4.3.2). Missing values were not imputed. The analyses were carried out on the original database, considering only the cases with information available for each variable (complete case analysis).

## 3 Results

### 3.1 Prevalence and demographic characteristics

The BIGAN database analyzed contained information on 1 128 498 patients from Aragon between 2019 and 2023, of which 51.1% were women. The mean age of the population with HF was 79.8 years (SD = 13.7), with a mean time since diagnosis of 6.6 years (SD = 5.9). During that period, a total of 30 677 patients were diagnosed with HF in this region, corresponding to a total prevalence of 2.7%. The total prevalence observed was higher in women than in men (2.8% vs. 2.6%), but higher in men in all age groups analyzed. Prevalence increased progressively with age, ranging from 0.2% in patients <45 years to 14.6% in those aged >75 years. The latter group accounted for the majority of patients with HF (*n* = 22 166; 72.3%), most of whom were women (*n* = 13 122) (Table 1).

**Table 1:** Study population and patients with heart failure (HF) by sex and age.

Age, years	18–44	45–55	55–65	65–75	>75	Total
Study population						
Men, *n* (%)	213,308 (18.9)	114,395 (10.1)	96,347 (8.5)	67,091 (6.0)	61,187 (5.4)	552,328 (48.9)
Women, *n* (%)	208,799 (18.5)	108,437 (9.6)	95,368 (8.5)	73,378 (6.5)	90,188 (8.0)	576,170 (51.1)
Total, *n* (%)	422,107 (37.4)	222,832 (19.7)	191,715 (17.0)	140,469 (12.5)	151,375 (13.4)	1,128,498
Population with HF						
Men, *n* (prevalence %)	373 (0.2)	559 (0.5)	1,600 (1.7)	2,760 (4.1)	9,044 (14.8)	14,336 (2.6)
Women, *n* (prevalence %)	321 (0.2)	340 (0.3)	747 (0.8)	1,811 (2.5)	13 122 (14.6)	16,341 (2.8)
Total, *n* (prevalence %)	694 (0.2)	899 (0.4)	2,347 (1.2)	4,571 (3.3)	22 166 (14.6)	30,677 (2.7)

### 3.2 Comorbidities

The most prevalent comorbidities associated with HF were: HTN (*n* = 19 475; 63.5%), dyslipidemia (*n* = 14 991; 48.9%), obesity (*n* = 12 106; 39.5%), CKD (*n* = 10 875; 35.4%), atrial fibrillation (*n* = 10 440; 34.0%), T2DM (*n* = 9,901; 32.3%), and iron deficiency anemia (*n* = 9,384; 30.6%) (Table 2).

**Table 2:** Associated comorbidities in patients with heart failure.

Associated comorbidities	*n* (%)
HTN	19,475 (63.5%)
Dyslipidemia	14,991 (48.9%)
Obesity	12,106 (39.5%)
CKD	10,875 (35.4%)
Atrial fibrillation	10,440 (34.0%)
T2DM	9,901 (32.3%)
Iron deficiency anemia	9,384 (30.6%)
Vascular disease	6,828 (22.3%)
Thyroid disorders	5,874 (19.2%)
Smoking	3,781 (12.3%)
Ischemic heart disease	4,021 (13.1%)
COPD	3,970 (12.9%)
Asthma	2,979 (9.7%)
Cirrhosis	1,385 (4.5%)
Alcoholism	741 (2.4%)

CKD, chronic kidney disease; COPD, chronic obstructive pulmonary disease; HTN, hypertension; T2DM, type 2 diabetes mellitus.

### 3.3 Laboratory parameters

Notable among the laboratory test results were the median [IQR] eGFR of 60.2 mL/min/1.73 m^2^ [43.0 78.5] and the medians [IQR] BNP and proBNP of 340.5 pg/mL [163.6 993.6] and and 1,160.2 pg/mL [397.1 2,939.0], respectively, both showing skewed distributions, Other laboratory parameters of interest, such as pCr, UACR, hemoglobin, platelets, and ferritin are described in Table 3.

**Table 3:** Laboratory test results for patients with heart failure.

Parameter (unit)	Mean (SD)
eGFR (mL/min/1.73 m^2^)	60.3 (22.4); 60.2 [43.0 78.5]
pCr (mg/dL)	1.2 (0.7); 1.0 [0.8 1.3]
UACR (mg/g)	108.5 (468.0); 16.6 [6.1 51.6]
BNP (pg/mL)	1,079.6 (2,322.1); 340.5 [163.6 993.6]
proBNP (pg/mL)	2,917.2 (6,167.0); 1,160.2 [397.1 2,939.0]
Hemoglobin (g/dL)	13.2 (1.9); 13.3 [12.0 14.5]
Platelets (10⁹/L)	217.7 (75.9); 207.0 [168.0 255.0]
Ferritin (ng/mL)	218.6 (314.5); 126.1 [58.0 262.1]
TSH (mIU/L)	2.5 (3.2); 2.0 [1.3 3.1]
AST (U/L)	25.6 (123.2); 21.0 [17.0 27.0]
ALT (U/L)	20.6 (34.3); 16.0 [11.3 22.8]
ALP (U/L)	88.9 (54.9); 79.0 [64.0 100.0]
HbA1c (% of total Hb)	6.2 (1.0); 5.9 [5.6 6.5]

UACR, urine albumin-creatinine ratio; ALP, alkaline phosphatase; ALT, alanine aminotransferase; AST, aspartate aminotransferase; BNP, B-type natriuretic peptide; eGFR, estimated glomerular filtration rate; HbA1c, glycosylated hemoglobin; pCr, plasma creatinine; TSH, thyroid-stimulating hormone.

Mean (SD); Median [IQR].

### 3.4 Pharmacological treatment

**Figure 1:**
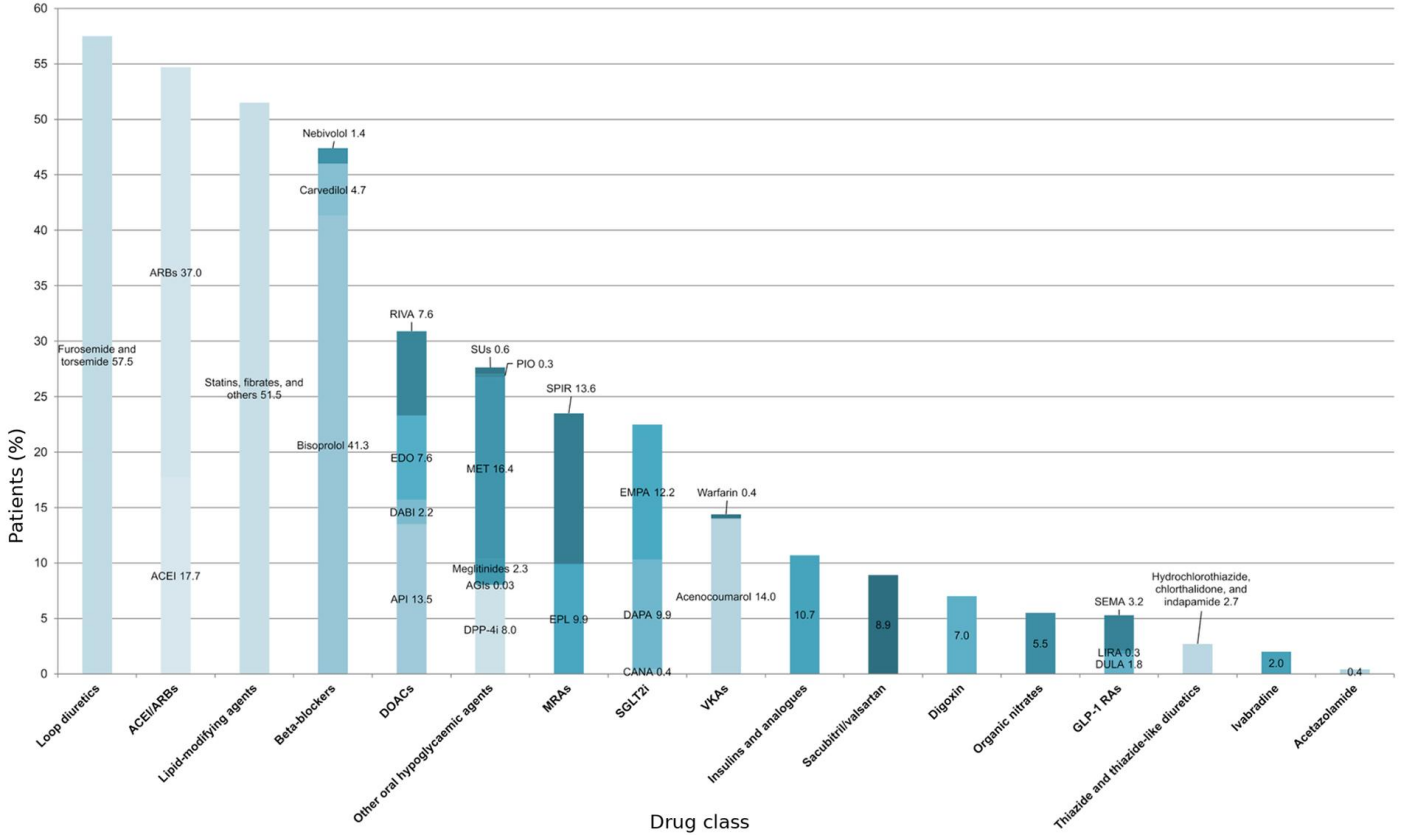
Pharmacotherapy in the study cohort. Horizontal bar chart depicting the proportion of patients prescribed each drug class and selected agents (*x*-axis, %). Totals exceed 100% because patients could be prescribed multiple medications. ACEI, angiotensin-converting enzyme inhibitors; AGIs, alpha-glucosidase inhibitors; API, apixaban; ARBs, angiotensin II receptor blockers; CANA, canagliflozin; DABI, dabigatran; DAPA, dapagliflozin; DOACs, direct oral anticoagulants; DPP-4i, dipeptidyl peptidase-4 inhibitors; DULA, dulaglutide; EDO, edoxaban; EMPA, empagliflozin; EPL, eplerenone; GLP-1 RAs, glucagon-like peptide-1 receptor agonists; LIRA, liraglutide; MET, metformin; MRAs, mineralocorticoid receptor antagonists; PIO, pioglitazone; RIVA, rivaroxaban; SEMA, semaglutide; SGLT2is, sodium-glucose cotransporter-2 inhibitors; SPIR, spironolactone; SUs, sulfonylureas; VKAs, vitamin K antagonists.

Analysis of the treatments received by the study population with diagnosed HF revealed that diuretics, cardiorenal drugs, and lipid-lowering agents were the most commonly prescribed. Loop diuretics were the most widely used medications (*n* = 17 631; 57.5%). Lipid-lowering agents were prescribed to 51.5% (*n* = 15 809) of patients with HF. Among beta-blockers (*n* = 14 554; 47.4%), bisoprolol accounted for 41.3% (*n* = 12 673), while carvedilol and nebivolol were less frequently prescribed (*n* = 1 457, 4.7%; and *n* = 424, 1.4%, respectively). In renin-angiotensin-aldosterone system regulation, ARBs were used in 37.0% (*n* = 11 352) of patients, ACEIs in 17.7% (*n* = 5 419), and the MRAs spironolactone and eplerenone in 13.6% (*n* = 4 171) and 9.9% (*n* = 3 052), respectively; sacubitril/valsartan accounted for 8.9% (*n* = 2 730). SGLT2is were indicated in 22.5% (*n* = 6 812) of patients (empagliflozin, *n* = 3 756, 12.2%; dapagliflozin, *n* = 3 034, 9.9%; and canagliflozin, *n* = 121, 0.4%). Metformin (*n* = 5 025; 16.4%), insulins and analogues (*n* = 3 288; 10.7%), dipeptidyl peptidase-4 (DPP-4) inhibitors (*n* = 2 453; 8.0%) and glucagon-like peptide-1 (GLP-1) receptor analogues (*n* = 1 567; 5.3%) were observed in metabolic comorbidity. In anticoagulation, the most commonly used were acenocoumarol (*n* = 4 413; 14.0%), apixaban (*n* = 4 139; 13.5%), rivaroxaban (*n* = 2 320; 7.6%), edoxaban (*n* = 2 342; 7.6%), and dabigatran (*n* = 684; 2.2%). Finally, digoxin was prescribed in 7.0% (*n* = 2 144) and ivabradine in 2.0% (*n* = 620) of patients with HF (Figure 1).

### 3.5 Level of care where diagnosis was made and resource utilization

**Figure 2:**
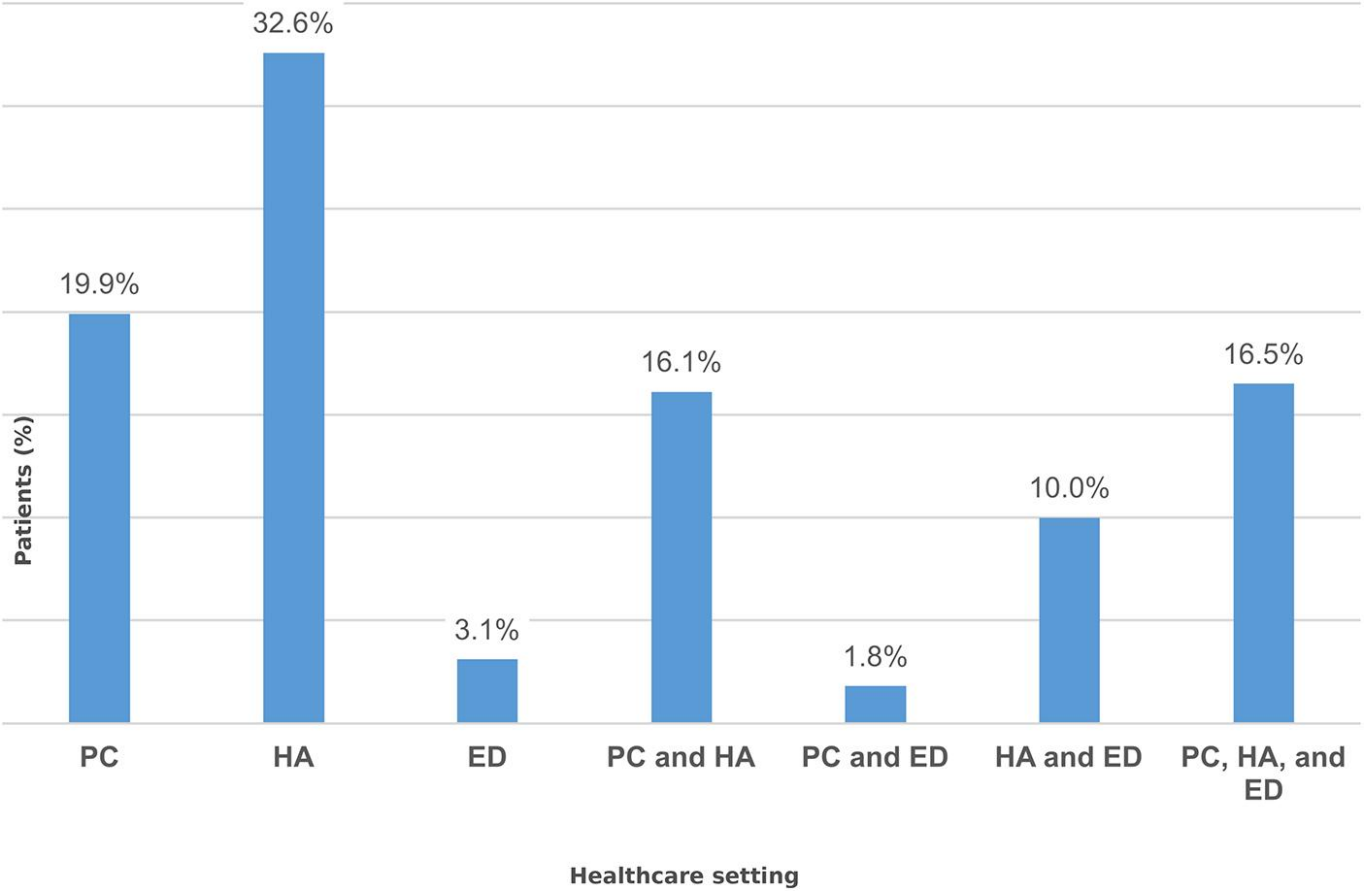
Distribution of heart failure diagnoses across the different levels of care in the public healthcare system. PC (diagnosis made exclusively in primary care), HA (diagnosis made exclusively during hospital admission), ED (diagnosis made exclusively in the emergency department), PC and HA (diagnosis shared between PC and HA), PC and ED (diagnosis shared between PC and ED), HA and ED (diagnosis shared between HA and ED), PC, HA, and ED (diagnosis shared among PC, HA, and ED).

The levels of care where HF was diagnosed between 2019 and 2023 in Aragon were classified as follows: diagnosis made exclusively in PC, ED, or HA; diagnosis made in PC and ED; diagnosis made in PC and HA; diagnosis made in ED and HA; and diagnosis made in PC, ED, and HA. The data show that the majority of HF diagnoses (almost one third) were made during HA. The second healthcare setting to exclusively diagnose HF was PC, with almost one in five diagnoses. In addition, almost 45% of HF diagnoses were made jointly between different levels of care, most notably the diagnoses made in PC, ED, and HA. ED was the healthcare setting with the lowest percentage of diagnoses made exclusively (Figure 2).

From the point of view of healthcare, a gradual increase in both ED visits and HAs due to HF was observed between 2019 and 2023. This increase was particularly notable in HAs, where the average annual growth increased more than fourfold over this period. ED visits also showed a steady rise, albeit with a smaller magnitude: approximately 45% over the 5-year period (Supplementary Table S3).

## 4 Discussion

### 4.1 Epidemiological context and prevalence

Over the past few decades, the burden of HF has increased steadily, with a 91.9% rise in the number of cases between 1990 and 2017 (1). It has an estimated prevalence in Spain of 1%–7% (3, 6, 7) and is one of the leading causes of HA in the Spanish population aged ≥65 years, accounting for around 3% of all HAs and 2.5% of healthcare expenditure (2, 3). This increasing burden is associated with high comorbidity, recurrent HAs, poorer quality of life, and high mortality (11, 12), driven by population ageing (6, 10) and factors such as T2DM (14–16), underlining the need for robust epidemiological data to guide decision-making.

The findings of this study indicate that the prevalence of HF in the Autonomous Region of Aragon between 2019 and 2023 was 2.7%. Overall prevalence was higher in women and increased progressively with age to over 14% in adults >75 years. These results are consistent with previous national studies. For example, the PRICE study, which analyzed a random sample between 2004 and 2005, reported a prevalence of HF of 6.8% in Spain, with a higher prevalence in women and in individuals aged >75 years (6). Differences between prevalence estimates from the aforementioned study and ours could be explained by methodological differences (random vs. total population sample) or actual changes in prevalence over time. Another study conducted in Catalonia (retrospective, observational, cohort study; 2010–2013) also showed a higher number of women with HF and an over-representation of HF in patients >75 years (20). In addition, a national, retrospective cohort study using the BIG-PAC database (2013–2019) estimated a total prevalence of 2.3%, again with a higher number of patients with HF >75 years compared to the rest of the age ranges. This analysis also evaluated prevalence by phenotype, observing a higher prevalence of HFrEF (49.3%) compared to HFpEF (38.1%), with variations across age groups. Overall, the prevalence observed in our study is consistent with the findings of this national study, which again shows that Aragon is representative of Spain as a whole (21). We also provide the prevalence by age group and sex, which gives a more comprehensive view of the demographic characteristics of HF. For example, in our study we observed that the age-specific prevalence in Aragon is higher in men in all ranges; however, due to the population structure, the global prevalence is higher in women. Both our study and the BIG-PAC analysis show that there are more women with HF among patients >75 years old, probably due to a survival bias, given that the female population is much higher than the male population in this age range; in fact, in our study, this does not translate into a higher age-specific prevalence in women.

### 4.2 Comorbidity and risk profile

In our Aragon cohort, the most frequent comorbidities were HTN, dyslipidemia, obesity, CKD, and T2DM. This profile is in line with findings from previous series, such as CaReMe Spain, which underlines the T2DM-CKD relationship (14) and is consistent with international analyses of the cardio-renal-metabolic axis (CaReMe-CKD) (22). CKD, which was highly prevalent in our cohort (35%), maintains a bidirectional association with HF within the cardiorenal axis, which complicates management and increases mortality (23). Furthermore, T2DM, present in nearly one-third of patients in our study, has been associated with an increased risk of HA for HF in large population-based studies (e.g., a Swedish cohort of 271 174 subjects) (24), and evidence synthesis links diabetes mellitus to an increased risk of HF onset or relapse as well as higher mortality in chronic HF (25, 26). For example, a recent population study in Catalonia (16) observed that 4.7% of individuals in the T2DM group were hospitalized for the first time for HF compared to 1.7% in the no T2DM group. These differences remained stable over the 13 years of follow-up, despite the introduction of new therapeutic options for metabolic control (including SGLT2is and GLP-1 receptor agonists). The main predictors of HA for HF in patients with T2DM were HTN, atrial fibrillation, ischemic heart disease, and CKD, in addition to advanced age, male sex, obesity, dyslipidemia, and overall comorbidity. These were also the most frequent comorbidities found in our study.

Atrial fibrillation and iron deficiency anemia, present in about one-third of HF patients in our cohort, have also been identified as common and clinically relevant in other studies. There is evidence that HF associated with atrial fibrillation has a worse prognosis (27), and intravenous iron replacement has been shown to reduce HA for HF after discharge and to provide a sustained benefit in chronic HF (28).

In relation to modifiable factors, HTN remains one of the main risk factors for the development of HF, as demonstrated by the Framingham study (29). Moreover, obesity, present in almost two out of five patients with HF in our study, has been associated in recent meta-analyses to an increased risk of HF, even in phenotypes defined as metabolically healthy (30), so weight control remains an important therapeutic objective.

### 4.3 Pharmacological management and guideline adherence

In this study, the pharmacological profile of HF patients was characterized by the use of loop diuretics in more than half of all cases. Similar prescribing rates have been described in other population studies, such as the SwedeHF Registry (31) and the CHAMP-HF Registry (32) (59% and 61%, respectively). However, it should be noted that loop diuretics were given a class I recommendation for relieving fluid retention and congestion in the 2023 update of the European Society of Cardiology (ESC) clinical practice guidelines, although they are not considered a prognosis-modifying therapy (5). In line with this, their use is intended for symptom control, and they have not been shown to reduce mortality; therefore, once euvolemia has been achieved, the dose should be reduced to the minimum effective level (or even discontinued) and the patient should be closely monitored, prioritizing the optimization of drugs that improve survival. The persistence of high or unnecessary doses despite euvolemia suggests therapeutic inertia in adjusting diuretic treatment in chronic HF (33).

Regarding the recommended treatments for HF, the clinical practice guidelines of the ESC and the American College of Cardiology recommend early, combined introduction of the “four pillars” in HFrEF (SGLT2is, ARNIs/ACEIs/ARBs and beta-blockers, plus MRAs) with rapid titration due to their proven impact on morbidity and mortality in patients with HFrEF (5, 34). In addition, SGLT2is (dapagliflozin and empagliflozin), a mainstay in the treatment of HFrEF, have also been shown to reduce the composite endpoint of cardiovascular death or HA for HF in HFpEF (35, 36).

This study reinforces descriptions at the national level in Spain: patients with HF in Aragon have a high pharmacological burden, with therapy being based on extensive use of beta-blockers and ACEIs/ARBs, in line with contemporary Spanish cohorts (7). However, recent Spanish registers show lower use of ARNIs and SGLT2is in vulnerable subgroups (37, 38). In HF with CKD, the prescription of both the latter decreases progressively as the eGFR decreases (37). In the study by Guzman-Carreras et al. (2024) (38) in patients >75 years with multiple pathologies, prescription at discharge was also low (SGLT2i, 28%; ARNI, 9.3%) and the use of SGLT2is was associated with fewer readmissions at 12 months and lower mortality (including in-hospital mortality) with no increase in adverse effects, suggesting that their underuse could worsen prognosis. Although the PATHWAYS-HF study (7) and the study by Escobar et al. (2022) (39) point to some recent improvement in this regard in Spain, significant gaps still persist (especially in the use of SGLT2i and ARNIs), which is consistent with our findings in Aragon. Camps-Vilaró et al. (2020) (40) estimated that in Spain, around 245 789 patients aged ≥45 years who meet DAPA-HF study (36) criteria for HF (HFrEF <40%, NYHA functional class II–IV, and eGFR ≥30 mL/min/1.73 m^2^) (approximately 41.3% of HF patients in that age group) would benefit from treatment with SGLT2is. Taken together, the gap between evidence and practice underscores the need to implement specific strategies (protocols for early treatment initiation, care pathways for optimization, and prescribing support in CKD and in patients >75 years) to improve adherence to guidelines and ensure sustained access to therapies with proven prognostic benefit (5, 39). Because ejection fraction and HF phenotype were unavailable, treatment rates reported here should not be interpreted as evidence of inappropriate prescribing at the individual level. Also, these figures represent aggregate prescribing at the 31 December 2023 cut-off and should not be understood as judgments about guideline adherence in specific patients.

### 4.4 Care pathways, diagnosis setting, and healthcare utilization

In relation to the level of care, one third of patients in our cohort were diagnosed during HA, making this the most frequent setting for the diagnosis of HF. This is consistent with recent studies where more than 50% of diagnoses were made during the hospital stay (41). PC accounted for one-fifth of diagnoses, underscoring its role in early detection. In Aragon, the Aragon Institute of Health Sciences has recently incorporated HF indicators into its Atlas of Potentially Avoidable Hospitalizations, highlighting the key role of effective outpatient care in reducing avoidable admissions for HF (42). However, in the UK health system, >40% of patients with HF had been seen in PC for cardinal symptoms in the 5 years prior to the first hospital record of the diagnosis (43), indicating lost opportunities to pre-empt diagnosis in this healthcare setting. Diagnosis exclusively in ED was rare (3.1%), probably because patients are immediately admitted to hospital or are followed up as outpatients after discharge from the ED. Joint or sequential participation of different levels of care was common: >42% of patients were diagnosed in care pathways that combined PC, HA, and/or ED, reflecting the complexity of the diagnostic process and the progressive nature of the disease. The pattern observed also suggests a predominantly reactive model focused on advanced phases. This makes it essential to redesign strategies for early diagnosis and optimize care pathways, and reinforces the need for effective coordination between levels of care to improve the identification of HF and promote a comprehensive approach. Shifting diagnostic confirmation to PC, relying on natriuretic peptide screening (44, 45), risk algorithms in medical records (46), and rapid referral pathways to HF units (45) could shorten the time to initiation of prognosis-modifying therapies, and thereby reduce HAs and avoidable costs (44, 47).

With respect to healthcare resource utilization, our study confirms an average annual increase in ED visits and admissions for HF between 2019 and 2023. This pattern is consistent with a previous national trend (2003–2015) towards increased HAs for HF and with the older age and comorbidity of the hospitalized patients described in those series (48, 49). In HF decompensations, appropriate outpatient transition services (structured follow-up after discharge, home nursing visits, HF clinics, and case management) reduce readmissions, and home visits and HF clinics also reduce mortality. Therefore, improving quality, coordination, and continuity of care between levels of care should help to reduce pressure on hospitals (50). In the same line, the Atlas of Potentially Avoidable Hospitalizations developed in Aragon quantifies the recent (2018–2020) burden of disease at 142 880 admissions for HF (about 1/547 people ≥40 years), which underlines the potential impact of optimizing outpatient pathways and inter-level pathways to reduce avoidable admissions (42).

### 4.5 Limitations

Our study has some limitations, including its cross-sectional observational design, which prevents us from establishing causal relationships, and our reliance on retrospective information from the healthcare system, which can lead to recording and underdiagnosis biases in patients with mild or asymptomatic HF. Likewise, the absence of differentiation in the database between HFrEF and HFpEF has therapeutic implications because the indication for ARNIs, for example, is mainly limited to HFrEF (51), so this diagnostic classification is needed for these drugs to be prescribed correctly Consequently, interpretation of prescribing patterns is limited; without ejection-fraction data or information on contraindications and clinical decision-making, we cannot assess the appropriateness of individual treatment decisions Moreover, the differences presented in the results are exclusively numerical and purely descriptive, so their statistical significance was not evaluated, and therefore they should be interpreted with caution. No imputation procedures were used to replace missing values, and only complete cases were included in the analysis, which could bias the estimates obtained. Nevertheless, the use of a large, representative database such as BIGAN helps mitigate these biases and strengthens the validity of the results obtained. Furthermore, the population of Aragon is representative of the population of Spain in terms of age, sex, and nationality/immigration status, which gives greater value to the study (21).

## 5 Conclusions

In this population-based study in Aragon, HF is confirmed as a major health concern that clearly worsens with age. HF frequently coexists with HTN, dyslipidemia, obesity, T2DM, CKD, atrial fibrillation, and anemia to create a complex cardio-renal-metabolic profile. We observed increasingly high healthcare resource utilization, with a predominance of hospitalization and use of ED services. The diagnosis is largely established in the hospital setting, with the participation of different levels of care, revealing an eminently reactive care pathway. Taken together, our findings reveal opportunities to promote earlier clinical suspicion starting in PC, optimize continuity of care, and reinforce the prescription and maintenance of therapies based on clinical practice guidelines. In this regard, the prescribing patterns suggest that SGLT2is and ARNIs have not yet been fully adopted, and the use of loop diuretics is widespread, presenting a clear opportunity for optimizing prognosis-modifying treatment and its titration. Identifying high-risk subpopulations, improving post-discharge transitions, and implementing integrated chronic care models (including pharmacotherapeutic optimization) could help to reduce decompensations and admissions and improve survival and quality of life.

## Data Availability

The data analyzed in this study is subject to the following licenses/restrictions: the data underlying this article cannot be shared publicly due to data ownership and confidentiality restrictions associated with the BIGAN database. The data can be made available to qualified researchers upon reasonable request to the corresponding author, subject to approval by the BIGAN data custodian and execution of a data-use agreement. Requests to access these datasets should be directed to jcromerovigara@hotmail.es.
