# Supplementary material for: Prevalence and characterization of heart failure in Aragon, Spain (ICAR study)
Source: Front Cardiovasc Med. 2026 Mar 18;13:1749081. doi: 10.3389/fcvm.2026.1749081 (PMC13038503; doi:10.3389/fcvm.2026.1749081)
Supplement: Supplementary file 1 [file Table1.docx]

**Supplementary Table** **1.** Clinical conditions assessed in heart failure patients and corresponding ICPC-2 codes.

| **Condition** | **ICPC-2 code(s)** |
| --- | --- |
| Hypertension | K86/K87 |
| Dyslipidemia | T93 |
| Chronic kidney disease* | U99.01 |
| Atrial fibrillation | K78 |
| Type 2 diabetes mellitus | T90 |
| Iron deficiency anemia | B80 |
| Obesity** | T82 |
| Vascular disease | K89/K90/K91/K92 |
| Thyroid disorders | T85/T86 |
| Tobacco use | P17 |
| Ischemic heart disease | K74/K76 |
| Chronic obstructive pulmonary disease | R95 |
| Asthma | R96 |
| Cirrhosis/other hepatic diseases | D97 |
| Chronic alcohol use | P15 |

*Among patients without a recorded diagnosis of chronic kidney disease (CKD)—in primary care (U99), hospital, or emergency records—we reviewed estimated glomerular filtration rate (eGFR) values or, if eGFR was not available, plasma creatinine from 1 Jan 2019 to 31 Dec 2023. In patients without a recorded CKD diagnosis in whom the eGFR could not be determined, we reviewed urinary albumin–creatinine ratio values for the same period. In both cases, CKD was established when two values supporting the diagnosis were recorded at least 3 months apart.

**Obesity includes both recorded diagnoses and cases classified based on the BMI value closest to 31 Dec 2023, as documented in the medical record.
