# Supplementary material for: Prevalence and characterization of heart failure in Aragon, Spain (ICAR study)
Source: Front Cardiovasc Med. 2026 Mar 18;13:1749081. doi: 10.3389/fcvm.2026.1749081 (PMC13038503; doi:10.3389/fcvm.2026.1749081)
Supplement: Supplementary file 2 [file Table2.docx]

**Supplementary Table** **2.** Medications assessed in heart failure patients and corresponding Anatomical Therapeutic Chemical (ATC) classification codes.

| **Medication** | **ATC code** |
| --- | --- |
| Insulins and analogues | A10A |
| Metformin | A10BA02 |
| Sulfonylurea | A10BB12 |
| Alpha-glucosidase inhibitors | A10BF |
| Pioglitazone | A10BG03 |
| Dipeptidyl peptidase-4 inhibitors | A10BH |
| Liraglutide | A10BJ02 |
| Dulaglutide | A10BJ05 |
| Semaglutide | A10BJ06 |
| Dapagliflozin | A10BK01 |
| Canagliflozin | A10BK02 |
| Empagliflozin | A10BK03 |
| Other blood glucose-lowering drugs, excluding insulins | A10BX |
| Warfarin | B01AA03 |
| Acenocoumarol | B01AA07 |
| Dabigatran etexilate | B01AE07 |
| Rivaroxaban | B01AF01/X06 |
| Apixaban | B01AF02 |
| Edoxaban | B01AF03 |
| Organic nitrates | C01DA |
| Ivabradine | C01EB17 |
| Thiazides | C03AA |
| Sulfonamides (thiazide-like diuretics) | C03BA |
| Sulfonamides (loop diuretics) | C03CA |
| Spironolactone | C03DA01 |
| Eplerenone | C03DA04 |
| Bisoprolol | C07AB07 |
| Nebivolol | C07AB12 |
| Carvedilol | C07AG02 |
| ACE inhibitors | C09AA |
| ACE inhibitors and diuretics | C09BA |
| ACE inhibitors and calcium channel blockers | C09BB |
| Angiotensin II receptor blockers | C09CA |
| Angiotensin II receptor blockers and diuretics | C09DA |
| Angiotensin II receptor and calcium channel blockers | C09DB |
| Angiotensin II receptor blockers, other combinations | C09DX |
| Lipid-modifying agents | C10 |
| Acetazolamide | S01EC01 |

ACE, angiotensin-converting enzyme.
