# Supplementary material for: Prevalence and characterization of heart failure in Aragon, Spain (ICAR study)
Source: Front Cardiovasc Med. 2026 Mar 18;13:1749081. doi: 10.3389/fcvm.2026.1749081 (PMC13038503; doi:10.3389/fcvm.2026.1749081)
Supplement: Supplementary file 3 [file Table3.docx]

**Supplementary Table** **3**. Emergency department (ED) visits and hospital admissions (HA) in patients with heart failure.

| **Year** | **ED visits** | **HAs** |
| --- | --- | --- |
| 2019 | 0.7 (1.3); 0.0 [0.0, 1.0] | 0.4 (0.9); 0.0 [0.0, 1.0] |
| 2020 | 0.6 (1.1); 0.0 [0.0, 1.0] | 0.4 (0.8); 0.0 [0.0, 0.0] |
| 2021 | 0.7 (1.3); 0.0 [0.0, 1.0] | 0.5 (0.9); 0.0 [0.0, 1.0] |
| 2022 | 0.9 (1.5); 0.0 [0.0, 1.0] | 1.6 (2.3); 0.0 [0.0, 2.0] |
| 2023 | 1.0 (1.6); 0.0 [0.0, 1.0] | 1.8 (2.7); 0.0 [0.0, 3.0] |

Mean (SD); Median [IQR]
